# Effects of α and β-adrenergic signaling on innate immunity and Porphyromonas gingivalis virulence in an invertebrate model

**DOI:** 10.1080/21505594.2022.2123302

**Published:** 2022-09-19

**Authors:** Renata Mendonça Moraes, Maíra Terra Garcia, Fabio Stossi, Patrícia Pimentel de Barros, Juliana Campos Junqueira, Ana Lia Anbinder

**Affiliations:** a Institute of Science and Technology, Biosciences and Diagnosis Department, São Paulo State University (Unesp), São José dos Campos, SP, Brazil; b Department of Molecular and Cellular Biology, Baylor College of Medicine, Houston, Texas, USA; c GCC Center for Advanced Microscopy and Image Informatics, Houston, Texas, USA; d Multicampi School of Medical Sciences, Federal University of Rio Grande do Norte (UFRN), Caicó, RN, Brazil

**Keywords:** *Galleria mellonella*, *Porphyromonas gingivalis*, adrenergic signalling, invertebrates, innate immunity

## Abstract

To investigate the role of adrenergic signalling (AS) in the host immune response and *Porphyromonas gingivalis* virulence, we compared norepinephrine (NE) and isoproterenol (ISO) responses in *Galleria mellonella*. *P. gingivalis* infection was evaluated by survival; humoral immune responses (i.e. melanization and *cecropin* and *gloverin* mRNA expression); cellular immune responses (i.e. haemocyte count, nodulation by histology); and *P. gingivalis* recovery (CFU/mL). *P. gingivalis* was cultivated in the presence of ISO (PgISO) or NE and injected into the larvae for survival evaluation. Finally, we co-injected ISO and PgISO to evaluate the concomitant effects on the immune response and bacterial virulence. None of the ligands were toxic to the larvae; ISO increased haemocyte number, even after *P. gingivalis* infection, by mobilizing sessile haemocytes in a β-adrenergic-specific manner, while NE showed the opposite effect. ISO treatment reduced larval mortality and the number of recovered bacteria, while NE increased mortality and showed no effect on bacterial recovery. ISO and NE had similar effects on melanization and decreased the expression of cecropin. Although co-cultivation with NE and ISO increased the gene expression of bacterial virulence factors *in vitro*, only the injection of PgISO increased larval death, which was partially reversed by circulating ISO. Therefore, α- and β-adrenergic signalling had opposite effects after *P. gingivalis* infection. Ultimately, the catecholamine influence on the immune response overcame the effect of more virulent strains. The effect of AS directly on the pathogen found *in vitro* did not translate to the *in vivo* setting.

## Introduction

Stress contributes to the development and progression of various diseases due to the activation of several responses, including those of the sympathetic nervous system (SNS). SNS acts through α- and β-adrenergic receptors (AR), which are activated by the catecholamines epinephrine and norepinephrine (NE). Periodontitis, a chronic inflammatory disease of the supporting tissues of teeth, and the main cause of tooth loss in adults worldwide , is affected by SNS on its two pathogenesis pillars: host immune response and the microbiota [1,2].

Regarding the host response, the activation of the β-AR signalling leads to an increase in bone loss in rats with periodontitis [3], resulting in a pro-inflammatory profile [4,5] and decreased periodontal fibroblast proliferation [6]. Conversely, the blockage of the SNS by sympathectomy or propranolol (a β-AR blocker) inhibits osteoclastic differentiation and stress-induced interleukin (IL)-1β, IL-6, and IL-8 release, resulting in decreased bone loss [5,7–9].

In the microbiota, catecholamines have been shown to exhibit *in vitro* activity in some microorganisms. Epinephrine and NE interfere with the growth of some periodontopathogenic bacteria such as *Fusobacterium nucleatum* and *Tannerella forsythia* [10–14], while also increasing the virulence of other bacteria such as *Porphyromonas gingivalis*, the main pathogen associated with chronic periodontitis [15,16,17]. NE increases the expression of gingipain (*rgpb*), an important virulence factor of *P. gingivalis* [16,17]. This action can be reversed by the administration of propranolol, suggesting that a catecholaminergic receptor is expressed in the pathogen [18]. Although the current literature shows a direct action of catecholamines on *P. gingivalis*, the high concentrations used in these studies are not comparable to the concentrations in physiological or stress conditions, making the translation from bench to bedside a challenge.

Even though *in vitro* studies show the potential influence of adrenergic signalling directly on the main pathogen of periodontitis [16–18], and studies in rodents and humans highlight the deleterious impact of stress in periodontal disease, the complexity of the disease and the models used make it virtually impossible to study the SNS response alone. Thus, less complex models that still present an analogous response to bacterial infections, such as invertebrate models [19], are ideal to reveal the impact of the adrenergic system (AS) on the host immune response and pathogen virulence separately .

*Galleria mellonella*, a lepidopteran insect, has been widely used to study pathogen/host interactions owing to its many advantages. In the larval stage, it can be maintained at 37 °C, a temperature ideal for most human pathogens; it is also easy to handle, allows for the controlled inoculation of microorganisms, and its immune system resembles that of rodents and humans. Previous studies have shown that *G. mellonella* is a useful model for studying *P. gingivalis* infection [20–24]. Moreover, this insect produces octopamine (OCT), a hormone that structurally resembles NE [25,26].

Thus, to better understand the effects of the AS in the host immune response and the microbiota, specifically *P. gingivalis*, we investigated the systemic effects of isoproterenol (ISO) and NE at different concentrations (from low to high concentrations) in *P. gingivalis* infection outcomes using *G. mellonella*. Furthermore, we investigated the direct impact of ISO/NE on *P. gingivalis* virulence to verify the importance of SNS action on the bacteria versus that on the model’s (*G. mellonella*) immune system.

## Results

### ISO, NE, and OCT toxicity and cellular immune profiling

In *Galleria mellonella*, there was no to very limited toxicity across all tested concentrations, over a 5-day period, as compared with the PBS control group, for all three compounds (S1 appendix A – C).

**Figure 1.**
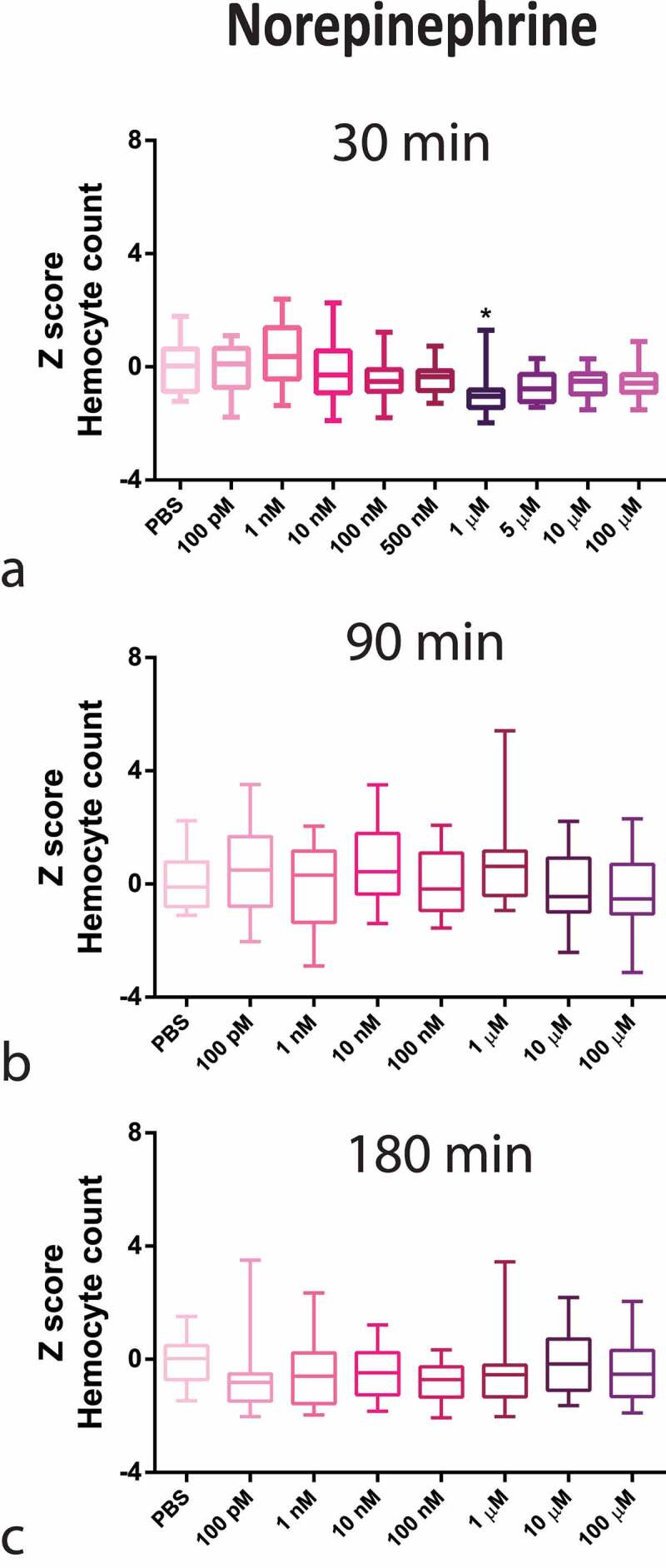
Z-Score-Normalized haemocyte counts at 30 (a), 90 (b), and 180 (c) minutes post-injection of several norepinephrine (NE) concentrations in *G. mellonella* larvae. Box plots represent z-normalized haemocyte counts for n = 20 larvae per concentration. NE decreases the number of circulating haemocytes at 1 µM (Kruskal–Wallis test, p < 0.0001) after 30 minutes. * Significantly different (p < 0.05) compared with the phosphate-buffered saline (PBS) group.

**Figure 2.**
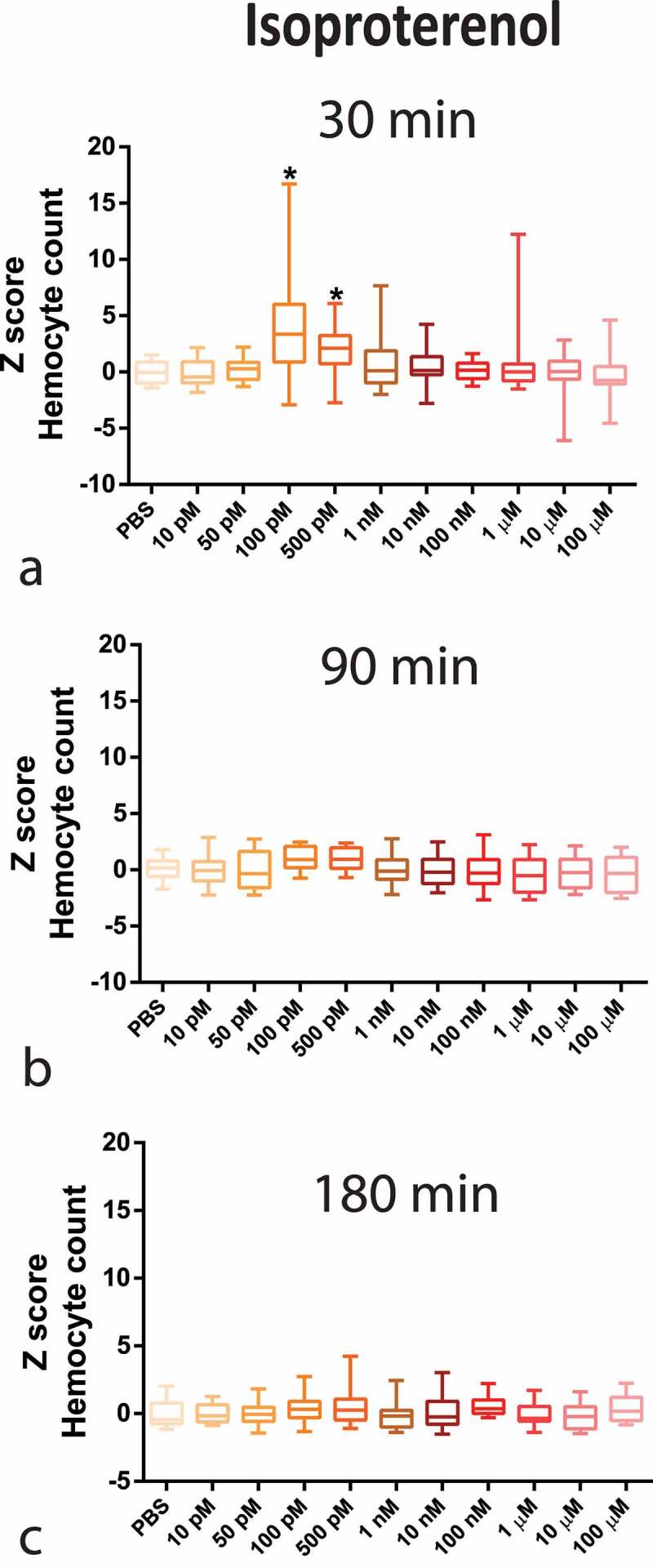
Z-Score-Normalized haemocyte counts at 30 (a), 90 (b), and 180 (c) minutes post-injection of a wide concentration range of isoproterenol (ISO) in *G. mellonella* larvae. Box plots represent z-normalized haemocyte counts for n = 20 larvae per concentration. ISO increases the number of haemocytes at 100 pM and 500 pM (Kruskal–Wallis test, p < 0.0001) after 30 min. * Significantly different (p < 0.05) compared with the phosphate-buffered saline (PBS) group.

Regarding circulating haemocytes, NE caused a decrease in haemocyte counts at a high concentration (1 µM), peaking at 30 min (Figure 1a). In contrary, ISO caused an increase in haemocyte count, with an inverse U-shaped concentration–response curve, peaking at 100 pM also at 30 min (Figure 2a). Interestingly, the results with 100 pM ISO were similar to the OCT endogenous hormone response (S2 appendix).

The complete time–concentration matrix for ISO and NE (Figure 1 and Figure 2) highlights the transient time response (30 min peak) to adrenergic stimuli with a narrow window of concentration dependency, indicating a highly tunable response.

### Systemic influence of adrenergic signalling (NE and ISO) in *G. mellonella* survival during *P. gingivalis* infection

#### ISO and NE influence on survival of *G. mellonella* during *P. gingivalis* infection

**Figure 3.**
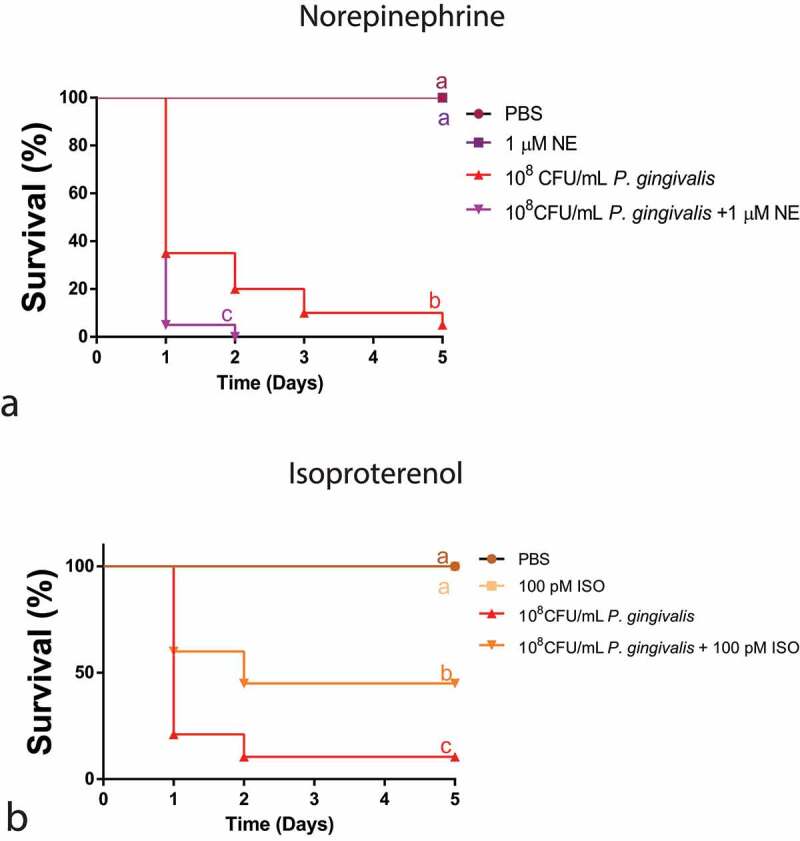
(a) Survival curve over 5 days of concomitant injection of 1 µM NE and 10^8^ CFU/mL *P. gingivalis* (n = 20 larvae per group, Kaplan–Meier plot, Log-rank [Mantel-cox] p = 0.0109). (b) Survival curve after concomitant administration of 100 pM ISO and *P. gingivalis* (n = 20 larvae per group, Kaplan–Meier plot, log-rank [Mantel-cox] p = 0.0099). Same lower-case letters close to the graph lines/bars represent absence of significant differences.

*P. gingivalis* injection killed 92.5% of larvae in 5 days, whereas co-injection with NE led to 100% larval death in only 2 days; this difference being statistically significant (Figure 3a). Opposite to NE response, there was a pro-survival effect of 100 pM ISO on the larvae co-injected with *P. gingivalis*, where the proportion of dead larvae was reduced to 45% by ISO after 5 days (Figure 3b).

#### *P. gingivalis* recovery from the haemolymph

**Figure 4.**
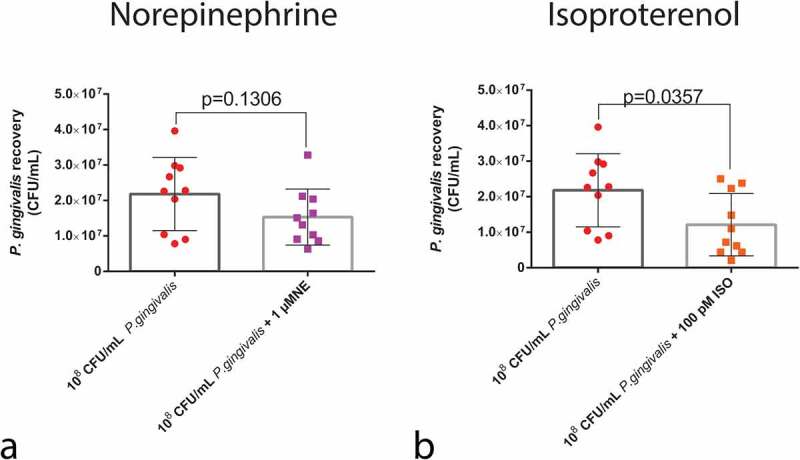
*P. gingivalis* recovery from the haemolymph of larvae infected with *P. gingivalis* ±1 µM NE (n = 10 pools of three larvae per group, mean and standard deviation, *t*-test, p = 0.1306) measured as CFU/mL (a) and *P. gingivalis* ±100 pM ISO (n = 10 pools of three larvae per group, mean and standard deviation, *t*-test, p = 0.0357) (b).

To investigate if the phenotypes observed in the survival curve analysis were correlated with bacterial load in the insect haemolymph, we measured the CFU/mL of *P. gingivalis* 30 min post-infection. NE treatment did not influence *P. gingivalis* recovery from the larvae haemolymph, since the *P. gingivalis* + NE group presented similar bacterial counts to the *P. gingivalis* control group (Figure 4a).

However, the larvae that received ISO + *P. gingivalis* had a reduction in the number of *P. gingivalis* cells recovered from the haemolymph (Figure 4b). These results highlight the fact that ISO treatment is capable of rapidly reducing the number of live bacteria post-infection.

#### Quantification of melanization and antimicrobial peptides

**Figure 5.**
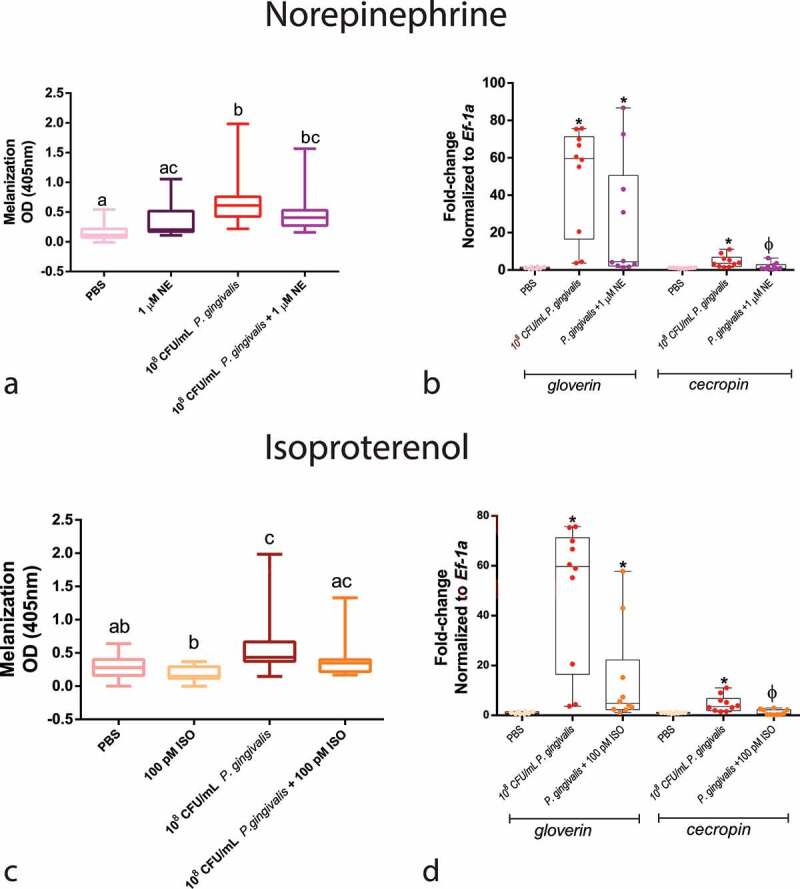
Humoral response of larvae to *P. gingivalis* infection. Colorimetric measurement of melanization for (a) NE (n = 20 larvae per group, Box plot, Kruskal–Wallis test, p < 0.0001) and (c) ISO (n = 20 larvae per group, Box plot, Kruskal–Wallis test, p < 0.0001). qPCR analysis of *gloverin* and *cecropin* mRNA expression 24 h after infection and NE administration (b) (*gloverin*: n = 8 larvae for PBS group and n = 10 larvae for *P. gingivalis* +/- 1 µM NE; Kruskal–Wallis test, p < 0.0001; *cecropin* : n = 10 larvae for PBS and *P. gingivalis* and n = 9 for *P. gingivalis* + 1 µM NE, Kruskal–Wallis test, p = 0.0024). (d) *P. gingivalis* +/- ISO (*gloverin*: n = 8 for PBS group, and n = 10 for *P. gingivalis* and *P. gingivalis*+ 100 pM ISO groups, Box plot, Kruskal–Wallis test, p < 0.0001; *cecropin*: n = 10 larvae per group, Kruskal–Wallis test, p = 0.0024). Same lower-case letters close to the graph lines/bars represent an absence of significant differences. * Significantly different compared with the PBS group.

We colorimetrically measured the levels of larval melanization at 30 min and antimicrobial peptides (AMP) gene expression 24 hours post-injection to determine whether the changes in survival caused by NE/ISO were influenced by the humoral immune response. Regarding melanization, neither compound influenced it when compared with *P. gingivalis* alone group (Figure 5a,c). However, concerning AMP production, *P. gingivalis* infection increased the expression of *cecropin* and *gloverin* by 49 and 4.6-fold, respectively. Co-infection with both NE and ISO decreased the expression of *cecropin* when compared with the *P. gingivalis* group (Figure 5b,d).

#### Haemocyte density

**Figure 6.**
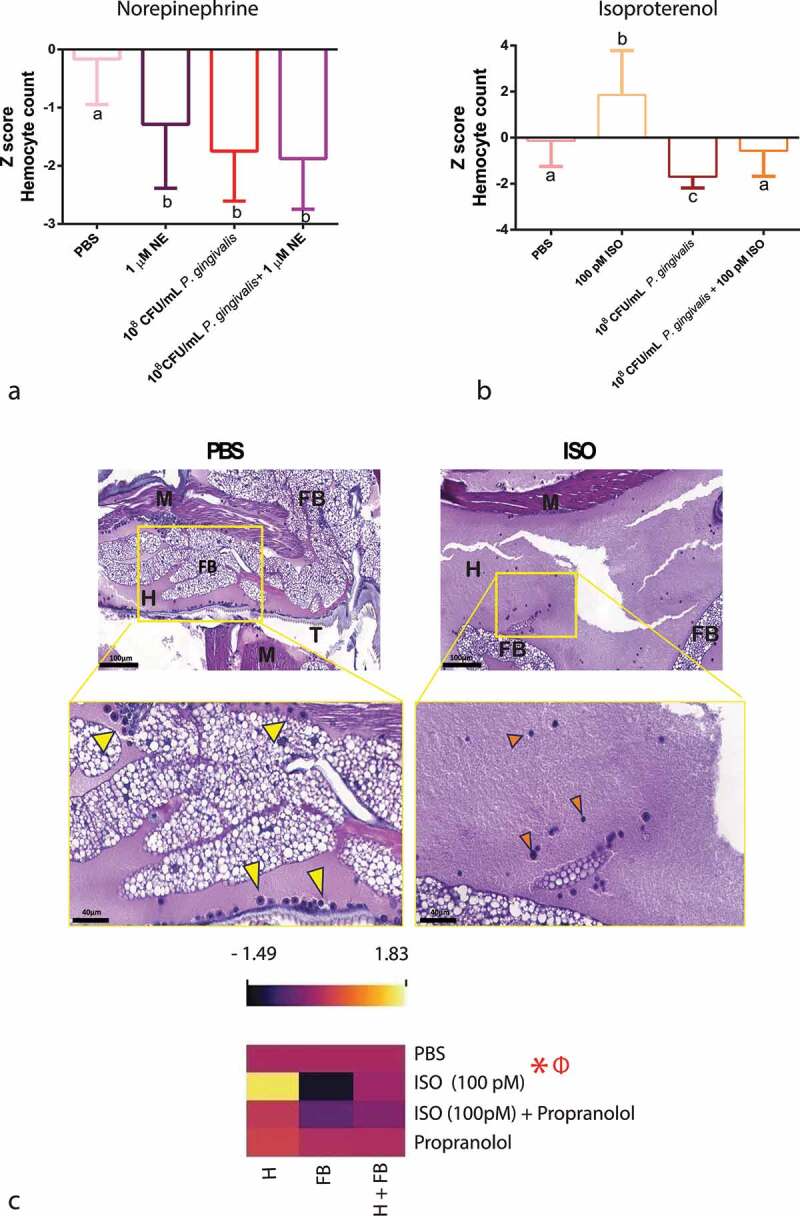
Haemocyte count. Upper panels: Z-score-normalized haemocyte counts measured after *P. gingivalis* ±1 µM NE compared to PBS group (n = 20 larvae per group, mean and standard deviation, ANOVA, p < 0.0001) (a) and ±100pM ISO (n = 20 larvae per group, mean and standard deviation, ANOVA, p < 0.0001) (b). Same lower case letters close to the graph lines/bars represent the absence of significant differences. Bottom panels: representative image of an H&E-stained sagittal section of PBS or ISO (100 pM) injected larvae. Image shows the sessile haemocytes adhering to the fat body (FB), muscle (M), and trachea (T) (yellow arrowhead) and circulating haemocytes in the haemolymph (H) (orange arrowhead) (scale bar: 100 µm in the upper images and 40 µm in the insets). (c) Z-score-normalized haemocyte counts from haemolymph and fat body/adjacent organs in larvae that received PBS or 100pM ISO ± propranolol [10 µM] (n = 20 larvae per group, heatmap, ANOVA, p < 0.0001). * Significantly different compared with the PBS group (on haemolymph). Φ Significantly different compared with the PBS group (on fat body/adjacent organs).

As shown earlier (Figure 1a), in the absence of infection, NE decreased the number of circulating haemocytes, which was also observed upon co-injection with *P. gingivalis* (Figure 6a), even though without reaching statistical significance from *P. gingivalis* alone group.

Differently, the larvae that received *P. gingivalis* together with ISO had a bacterial-induced decrease in haemocyte counts that was reversed to PBS levels (Figure 6b). Analysing circulating (in the haemolymph) and sessile haemocytes (from the fat body and adjacent organs – e.g. dorsal tube), from the same larvae, we demonstrated that the increase in circulating haemocytes corresponded to a comparable decrease in sessile haemocytes from the internal organs, clearly showing that ISO directly affects the release of sessile haemocytes (Figure 6c). This can also be seen in the histology sections (highlighted in the inserts of Figure 6) and has been quantified in the corresponding heatmap (Figure 6c). The β-specific release of haemocytes from internal organs was also observed for the endogenous hormone OCT (S3 appendix). We validated that the ISO/OCT-induced increase in circulating haemocytes was dependent upon the β-AR signalling by co-injecting the larvae with the β-AR-specific antagonist propranolol. As expected, propranolol completely blocked the ISO/OCT-induced increase in the circulating haemocyte count (Figure 6c, S3 appendix).

#### Nodule formation

**Figure 7.**
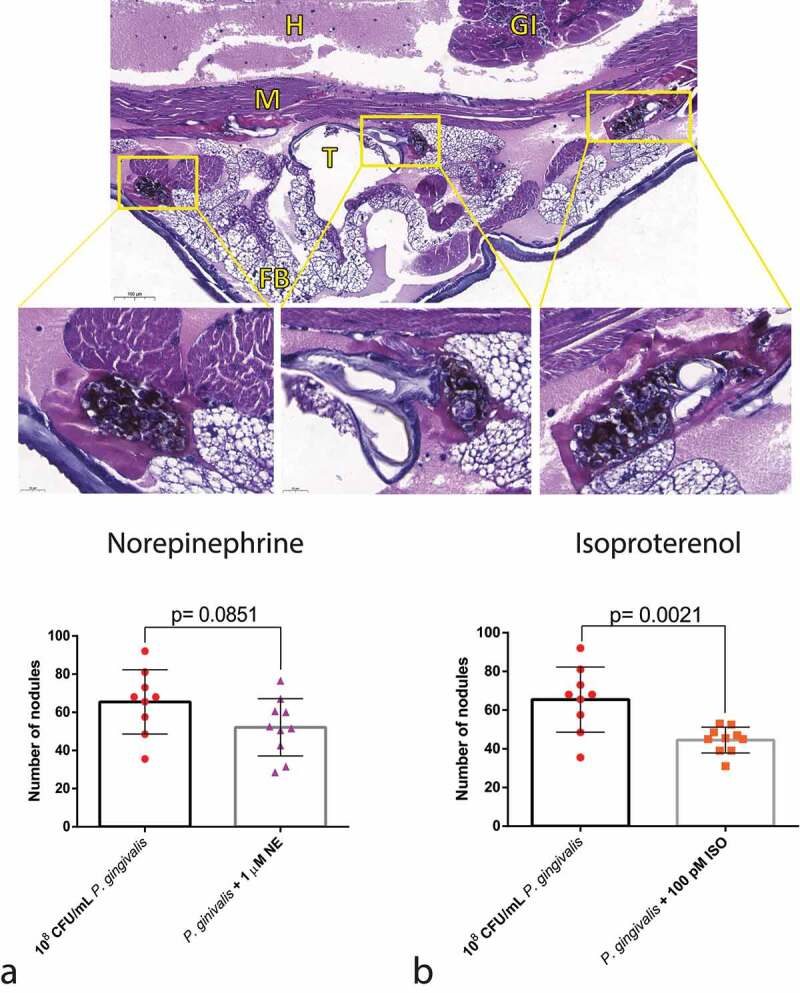
Effects of adrenergic signalling on nodulation. (Top) Representative figures of larvae injected with *P. gingivalis* after 30 min, showing nodules formed around the fat body, muscles, and trachea (H&E staining). GI = gastrointestinal tract, FB = Fat body, M = muscle, T = trachea, H = haemolymph. (Bottom) No differences observed between *P. gingivalis* and *P. gingivalis* +1 µm NE regarding number of nodules counted on histological sections (n = 10 larvae per group, three sections per larvae, mean and standard deviation, *t*-test, p = 0.0851) (a). Decrease in the number of nodules in the *P. gingivalis* +100 pM ISO (n = 10 larvae per group, 3 sections per animal, mean and standard deviation, *t*-test, p = 0.0021) (b).

As one of the hallmarks of the cellular response to bacteria is nodule formation, we performed a histological analysis of the infected larvae. NE did not lead to any difference in nodulation between the groups, in concordance with the *P. gingivalis* recovery assay (Figure 7a). On the other hand, ISO treatment decreased nodulation when compared with the *P. gingivalis* group (Figure 7b).

### Direct influence of adrenergic signalling (NE and ISO) on *P. gingivalis* virulence and infection outcome in *G. mellonella*

#### Co-culture of *P. gingivalis* with NE (α-AR)

**Figure 8.**
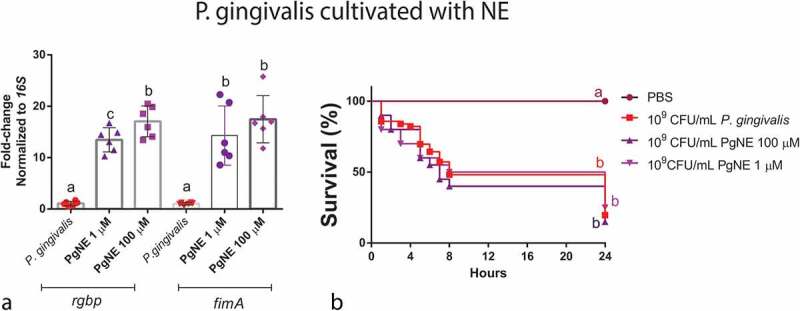
Direct action of adrenergic signalling on *P. gingivalis*. *rgpb* and *fimA* gene expression after co-culturing *P. gingivalis* ±1 µM or 100 µM NE (*rgbp*, n = 6 biological replicates per group, ANOVA, p < 0.0001; *fimA*, n = 6 biological replicates per group, mean and standard-deviation, ANOVA, p < 0.0001) (a). Co-culture of *P. gingivalis* with 1 µM or 100 µM NE had no effect on larvae survival 24 h after infection (n = 10 larvae for PBS group, n = 45 larvae for *P. gingivalis* group, and n = 20 larvae for PgNE 1 µM and PgNE 100 µM groups, Kaplan–Meier plot, log-rank [Mantel-cox] p = 0.0005) (b). Same lower-case letters close to the graph bars/lines represent an absence of significant difference.

Although co-culture with NE increased the gene expression of gingipain (*rgbp*) and fimbriae (*fimA*) *in vitro* (Figure 8a), it did not change the infection outcome *in vivo* (Figure 8b) leading to no difference in survival when compared to *P. gingivalis*.

#### Co-culture of *P. gingivalis* with ISO (β-AR)

**Figure 9.**
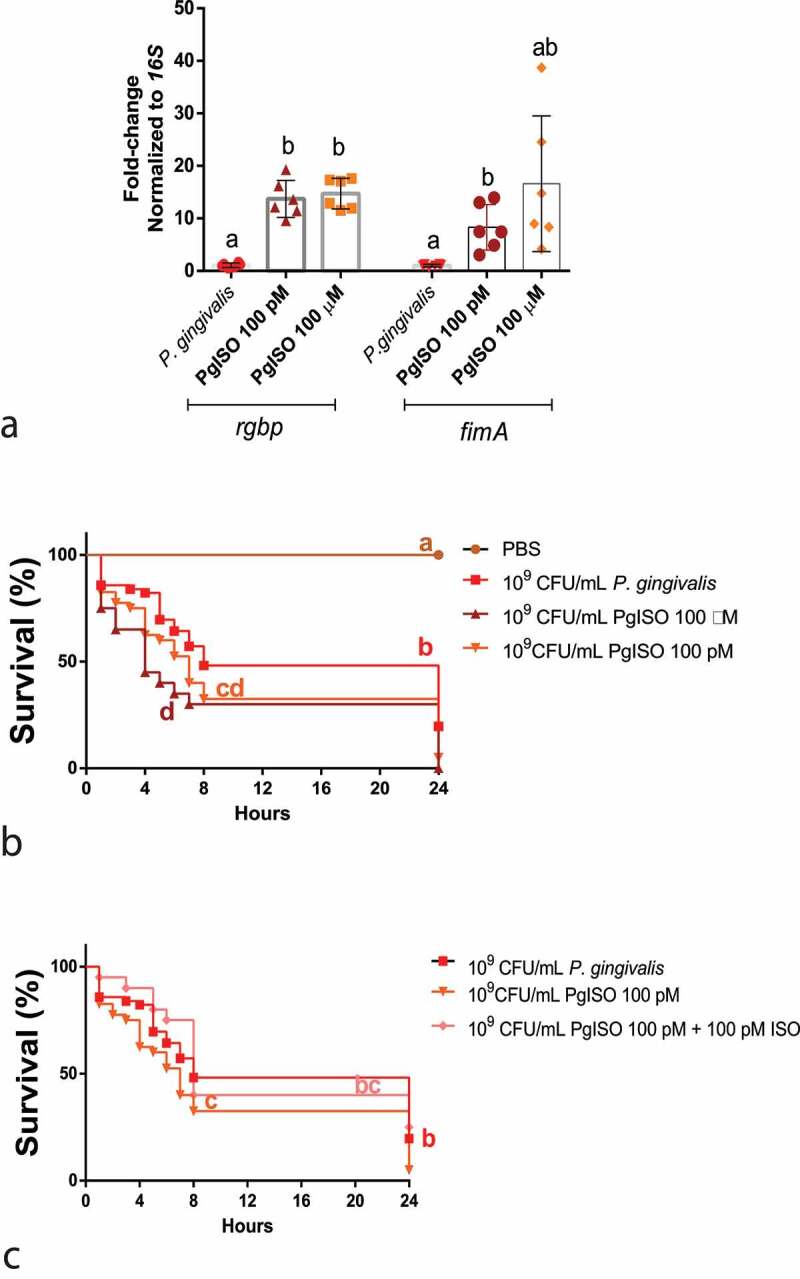
Direct action of β-adrenergic signalling on *P. gingivalis*. A) *rgbp* and *fimA* gene expression after co-culturing *P. gingivalis* ±100pM or 100 µM ISO (*rgbp*, n = 6 biological replicates per group, mean and standard deviation, ANOVA, p < 0.0001; *fimA*, n = 6 biological replicates per group, ANOVA, p = 0.0131) (a). Co-culturing *P. gingivalis* with 100 pM and 100 µM ISO increased the mortality of the larvae 24 h after infection (n = 10 larvae for PBS group, n = 45 larvae for *P. gingivalis* group, n = 20 for PgISO 100 pM and PgISO 100 µm and 100 pM ISO + PgISO 100 pM groups, Kaplan–Meier plot, log-rank [Mantel-cox] p < 0.0001) (b). The systemic administration of 100 pM ISO could partially reverse the increased PgISO 100pM phenotype (c). Same lower-case letters close to the graph bars/lines represent an absence of significant difference.

ISO influenced the gene expression of gingipain (*rgbp*) and fimbriae (*fimA*) *in vitro* and changed the phenotype *in vivo* (Figure 9a,b). ISO-grown *P. gingivalis* (PgISO) was more toxic to the larvae than *P. gingivalis* at both concentrations tested in the first 24 h post-injection, starting to kill the larvae within the first few hours (Figure 9b).

The interesting observation that ISO could behave differently if it was acting systemically on the larvae or directly on the bacterial environment prompted us to test whether the larvae pro-survival effects of circulating ISO could overcome the increased virulence of ISO-grown *P. gingivalis*. For this purpose, we co-injected *G. mellonella* with 100 pM ISO and 10^9^ CFU/mL ISO (100 pM)-grown *P. gingivalis* and observed them hourly for 24 h. As shown in Figure 9C circulating ISO was indeed able to partially reverse the toxicity of the more virulent PgISO, indicating that the systemic effects of the β-adrenergic signalling are sufficient to counteract the toxicity of bacteria with higher virulence.

## Discussion

In this study, we validated the adrenergic signalling response in *G. mellonella* haemocytes using known chemicals that bind to different ARs in higher organisms: ISO, a β-AR agonist, and NE, a preferential α-AR agonist. Haemocytes are insect cells analogous to neutrophils and macrophages that have been shown to express adrenergic-like receptors [27]. We showed that these receptors are functional, as the administration of either ligand influenced the cellular response in the larvae in a concentration- and time-dependent manner. Interestingly, the effect was time-restricted, peaking at 30 min post-injection (similar to OCT [28,29], supplementary figure S2) and returning to basal levels within the first hour. Since this is the first study to analyse the effects of ISO and NE on *G. mellonella* haemocytes, we can only correlate the findings with OCT observations. Therefore, the time points were chosen based on the literature for invertebrates regarding OCT, the insect counterpart of NE. OCT is released 15 min to 1 hour after diverse stimuli before returning to basal levels [28,29]. In addition, OCT and its downstream pathways (e.g PKA) influence haemocyte number and activity 20–30 minutes post injection of compounds or microorganism challenge [30–32]. Regarding concentration, some experiments showed increased haemocyte density at higher OCT concentrations (50–500 nM) and a decreased density at lower concentrations (5 nM) [30]. In contrast, an injection with 1 mM OCT increased haemocyte count in crickets [28], while OCT administration at ≤1000 pM mediated a transient upregulation of haemocyte count in shrimp (*Litopenaeus vannamei*) [33]. Different concentrations have been used to explain the observed OCT-dependent effects on immune function. In this study, we observed that OCT acted more like a β-AR agonist at lower concentrations (ISO-like) and as an α-AR agonist at higher concentrations (NE-like). In other invertebrates, β-AR signalling (ISO) has also been linked to increased haemocyte count [34,35], while α-AR signalling (NE) to decreased haemocyte count and increased death rate [36].

In invertebrates, an increase in circulating haemocytes has already been pointed out after stimulation and proposed to be due to the mobilization of these cells from the internal organs, especially the fat body, dorsal tube, and gastrointestinal tract [37]. In vertebrates, catecholamines also increase the number of circulating NK cells and granulocytes in response to stressors [38]. Herein, we show, for the first time, that the adrenergic response in *G. mellonella* is responsible for the swift (30 min) mobilization of haemocytes in a β-AR-specific manner. The specific intracellular pathways responsible for the mobilization and activation of haemocytes by β-AR are still under investigation. However, the protein kinase A and cyclic adenosine monophosphate pathways are known to be involved in haemocyte adhesion, one of the first steps in nodule formation [31,32,39] and phagocytosis [40–44].

Our results showed that, regarding *P. gingivalis* infection and virulence, the β-AR signalling was more prominent. *G. mellonella* was an excellent model to transition *in vitro* observations into *in vivo* scenarios, which aligns with the 3 R principles. Previous *in vitro* studies [11,16,18] using high concentrations of NE showed increased *P. gingivalis* virulence, as observed by the expression of virulence factors in our study. However, we did not observe any difference in PgNE compared to *P. gingivalis* infection regarding larval survival. In contrast, PgISO infection caused higher larval mortality. These findings could be strain-specific, as *Pseudomonas aeruginosa* H103 treated with 10 µM epinephrine increased bacterial virulence and decreased the survival of *G. mellonella* [45]. The increased mortality caused by the highly virulent ISO-treated *P. gingivalis* could be partially reversed by the systemic action of ISO on the immune response, thereby increasing the haemocyte density. This shows that the action of the AS on the immune response can overcome the higher bacterial virulence caused by the same agent.

We demonstrated that ISO action on survival was mainly related with the cellular response. On the humoral arm, we did not observe any influence of the ligands on melanization, as has already been shown for OCT [30]. Regarding the influence of infection and NE/ISO treatment on the gene expression of *cecropin* and *gloverin* (the two main AMPs against gram-negative bacteria) NE and ISO had some reducing effects on the mRNA levels of both peptides. It has become clear that stress influences the immune system by simultaneously upregulating some functions while downregulating others [41,46]. This balance is needed at the organismal level because over- or under-activation of immune reactions can damage the host. The decrease in AMP observed for both ligands could be explained by different mechanisms.

On the one hand, for ISO, this could be due to the decrease in the bacterial load leading to AMP reduction. On the other hand, the decrease observed in the NE group suggests that other AMPs, or additional factors, might be more important in the survival of *G. mellonella* upon *P. gingivalis* infection. These delicate balances between all innate immunity components may constitute the key response to survival and infection resolution. *Drosophila melanogaster* and *Sarcophaga peregrina* are not killed by *P. gingivalis*, whereas *G. mellonella* and silkworms suffer from lethal host damage caused by this pathogen [47], indicating that organismal differences in the innate immune system influence disease outcomes.

The *G. mellonella* model was limited by the inability for chronic exposure to bacteria and ligands, due to rapid adrenergic responses and lethal bacterial loads; therefore, we investigated the acute influence of α- and β-AR responses during *P. gingivalis* infection on innate immunity. The results clearly showed that the activation of β-AR during the initial phase of infection favours phagocytosis and bacterial clearing, preventing the death of the larvae.

## Conclusions

Herein, we validated the use of the *G. mellonella* model to investigate the role of adrenergic signalling during bacterial infection. We showed that the ligands binding to different ARs had opposite effects during *P. gingivalis* infection, with α-AR (NE) negatively affecting the course of infection, and β-AR (ISO) being protective for the larvae. The protective effect of ISO was mainly through the mobilization of sessile haemocytes to the haemolymph, where they decreased the bacterial load, leading to a more controlled infection. Finally, the systemic action of the ISO surpassed the negative action of more virulent bacteria, suggesting that the immune system can modulate itself to various stimuli, ultimately dictating the outcome of the infection.

## Materials and methods

### Galleria mellonella

Fifth instar larvae, reared in the laboratory of Microbiology and Immunology at the Institute of Science and Technology (ICT/UNESP/Brazil), weighing 0.15–0.25 g, were selected from a pool of healthy individuals that showed no signs of cuticle pigmentation. The larvae were stored at 25 °C [48] for one day before use when they were kept without food. There was no blinding of samples for the experiments and 20 larvae per group were used, unless otherwise stated.

### Bacteria growth conditions

ATCC *P. gingivalis* 33277 (Microbiologics^TM^) were activated in Brucella agar as the basis for the blood agar (5% defibrinated sheep blood), supplemented with 1% haemin and 1% menadione. The plates were incubated in an anaerobiosis jar with the aid of an anaerobiosis generator at 37 °C for 5–7 days. The bacteria were used between passages 4 and 8 for all experiments. Characteristic black colonies were harvested from the agar plates and suspended in PBS to 10^8^ CFU/mL (agar-grown bacteria). This suspension was used in the interaction experiments where *P. gingivalis* and ISO/NE were systemically injected in the last proleg of the larvae. For the experiments in which the bacteria were cultivated in the presence of the ligands (ISO/NE), colonies were transferred from agar to Brucella broth added to the ligands or PBS (control) and grown for 24 h in anaerobiosis (broth-grown bacteria). On the next day, samples were centrifuged at 5,000 rpm for 10 min and the pellet was resuspended in PBS to 10^9^ CFU/mL. The final suspensions were used for injecting the larvae.

### *G. mellonella* susceptibility to *P. gingivalis*

Bacterial suspensions (10 µL) were inoculated in the last left proleg of the *G. mellonella* larvae using micro syringes (Hamilton Inc., EUA) in different concentrations (10^7^ and 10^8^ CFU/mL for agar-grown bacteria; and 10^8^ and 10^9^ CFU/mL for broth-grown bacteria). After inoculation, the larvae were kept at 37 ºC, and survival was assessed daily for 5 days for agar-grown bacteria and hourly (up to 24 h) for broth-grown bacteria. At this point, the larvae were kept at 37 ºC because it is the ideal temperature for antimicrobial susceptibility testing of *P. gingivalis* [49], and it is well tolerated by the *G. mellonella* larvae [50,51].

### ISO, NE, and OCT toxicity and cellular immune profiling

To assess if the ligands were toxic for *G. mellonella*, a wide range of ISO (Isoproterenol hydrochloride, Sigma, I6504), NE (Noradrenaline tartrate, Sigma, N1100000), and OCT (Octopamine hydrochloride, Sigma, O0250) concentrations were injected into the last right proleg of the larvae. Concentrations were selected based on serum levels of NE in humans and OCT in insects during physiological, stress and pathological (pheochromocytoma) states [16–18,29,30,52,53]. The larvae were inoculated with 10 µL of ISO/NE/OCT or PBS, which served as the negative control. After injection, the larvae were kept at 37 ºC, and the survival was noted every day for 5 days. In a separate set of larvae, after 30, 90 and 180 min incubation at 37 °C, the haemolymph was bled through an incision on the last left proleg, diluted 1:100 in IPS buffer (2% NaCl; 0.1 M glucose; 30 mM sodium citrate; 26 mM citric acid and 10 mM EDTA) and the haemocytes were counted in a Neubauer chamber. This procedure was performed to temporally assess the influence of treatments on haemocyte density.

### ISO, NE, and OCT influence on the outcomes of *P. gingivalis* infection in *G. mellonella*

After concentration screening (see results section- Figure 1 and Figure 2), we chose 100 pM for ISO and 1 µM for NE with a 10^8^ CFU/mL of *P. gingivalis* for the subsequent experiments. Larvae were dived into six groups: PBS, *P. gingivalis*, 100 pM ISO, 1 µM NE, *P. gingivalis* +100 pM ISO, and *P. gingivalis* +1 µM NE. The insects were injected with 10 µL of PBS, ISO, or NE on the right last proleg, and immediately after they were injected 10^8^ CFU/mL of *P. gingivalis* or PBS in the last left proleg. The larvae were incubated at 37 °C for 5 days and survival curve was determined daily. Other experimental readouts for the interaction studies were measured 30 min after the inoculation, except for the AMPs gene expression that was performed 24 h later. Additional two sets of larvae were bled to collect haemolymph for melanization and haemocyte density evaluation.

### *P. gingivalis* recovery from the haemolymph

To analyse the possible influence of the ligands on bacterial load, the larvae were inoculated with 100 pM ISO, 1 µM NE, or PBS (control); and immediately infected with 10^8^ CFU/mL of *P. gingivalis*. The insects were then incubated at 37 °C for 30 min. The time period chosen was based on the transient action of the ligands on the haemocyte count and previous experience of our group, wherein the amount of bacteria naturally decreased after 3 h of infection [54]. Haemolymph from three larvae/group was collected by an incision in the last proleg and pooled, serially diluted to 1:10,000, 100 µL of the final dilution was plated on supplemented Brucella blood agar and incubated in anaerobiosis for 7 days. The characteristic black colonies were manually counted and expressed in CFU/mL. This procedure was performed with 10 pools of 3 larvae per group (total of 30 larvae per group).

### Quantification of melanization

A 20 µL of haemolymph from each larva was collected into pre-chilled Eppendorf tubes (30 min at −20 °C) as described above and centrifuged at 12,000 rpm for 10 min at 4 ºC. A 10 µL of the supernatant was diluted in 40 µL of IPS in a 96-well plate. The plate was incubated at RT for 5 min, and then it was read at 405 nm using a spectrophotometer.

### *G. mellonella* antimicrobial peptides (AMP) gene expression using RT-qPCR

Ten larvae per group were stored at −80 ºC, 24 h after injection of PBS, *P. gingivalis, P. gingivalis* +100 pM ISO and *P. gingivalis* +1 µM NE. The whole larvae were then macerated in liquid nitrogen using pestle and mortar. The powder was resuspended in 1 mL TRIzol® (ThermoFisher Scientific, 15,596,026), the RNA was isolated according to the manufacturer’s protocol and its concentration was measured using a NanoDrop 2000 Spectrophotometer. Only RNA samples with a 260/280 ratio of 1.8–2.0 and 230/260 ratio above 1.8 were used in reverse transcription. A 1,000 ng of the extracted RNA was treated with RQ1 RNase-Free DNase (Promega, M6101) and transcribed to cDNA using the GoScript™ Reverse Transcription Mix, Random Primers (Promega, A2801). Two reference genes for *G. mellonella*, *β-actin*, and *Ef-1a*, were run in all experimental groups. The obtained results were analysed at https://heartcure.com.au/, and the most stable gene was selected as the reference gene, which was *Ef-1a*. The primer sequences used are shown in Table 1. For the RT-qPCR reactions, GoTaq® qPCR Master Mix kit (Promega, A6002) was used. The reactions were performed in duplicate wells in a StepOnePlus™ Real-Time PCR System (Applied Biosystems, Foster, CA, USA), consisting of 5 μL GoTaq® qPCR Master, 0.3 μL CXR, 0.5 μM primers (final concentration), and target cDNA (2 µL of 1:10 diluted cDNA, final concentration-10 ng), supplemented with RNAse-free ddH_2_O to a final volume of 10 μL. The cycling parameters for the amplification reactions were 50 °C 20 s, 95 °C 3 min; 95 °C 20 s, 60 °C 1 min for 40 cycles; and 95 °C 15 s, 60 °C 1 min, 95 °C for 15 s. The level of gene expression was calculated by applying the 2 ^−ΔΔCT^ method.

**Table 1.** Genes investigating *P. gingivalis* virulence and *G. mellonella* immune defense.

Gene	Forward sequence	Reverse sequence	Amplicon size (base pairs)	Reference
***Porphyromonas gingivalis***				
*16S*	CGCCAACCTTTGAATCATTCCTT	TGGTGTCTGATCATGTTTCCAAGA	137	Azelmat et al., 2015 [55]
*FimA*	TTCTTGTTGGGACTTGCTGC	TCATCGCCAACTCCAAAAGC	140	Designed with primer3 based on GenBank:D17795.1
*Rgbp*	ACCATCGTCAGAGAGCGTAG	TACACTTGTCCCGACCGAAA	118	Designed with primer3 based on GenBank: 29,256,649
***Galleria mellonella***				
*ef1a*	CTCTGTTAAGGAGTTGCGCC	TGGCAATCGAGTACAGGTGT	147	Designed with primer3 based on GenBank: AF423811.1
*β-actin*	ACAGAGCGTGGCTACTCGTT	GCCATCTCCTGCTCAAAGTC	104	Rossoni et al., 2017 [56]
*gloverin*	AGATGCACGGTCCTACAG	GATCGTAGGTGCCTTGTG	93	Barros et al., 2019 [57]
*cecropin*	CTGTTCGTGTTCGCTTGTGT	GTAGCTGCTTCGCCTACCAC	158	Barros et al., 2019[57]

### Nodule quantification by histology

For histological analysis, 10 larvae from the groups *P. gingivalis*, *P. gingivalis* +100 pM ISO, and *P. gingivalis* +1 µM NE were used. Thirty minutes post-injection, the larvae were frozen at −20 °C for 10 min, injected with 100 µL of Bouin’s aqueous solution (Sigma, HT10132) in the last proleg for fixation, and kept at 4 °C for 72 hours. After that, the larvae were stored in 70% ethanol for 48 hours. For processing, the region between the last and second proleg was separated and hemisectioned, after which the pieces were routinely processed for paraffin embedding and haematoxylin & eosin staining. Three sections, 20 µm distant from each other per larvae, were evaluated in a brightfield microscope using a 20× objective. Structures containing more than 4 haemocytes arranged around a melanized area were considered a nodule. The number of nodules in the halves on the same histological plane was averaged and the values of the three sections were summed for statistical analysis.

### Haemocyte density in the haemolymph and fat body/adjacent organs

Haemocytes can be free in the haemocoel in the haemolymph (circulating haemocytes) or in the surface of internal organs such as the fat body and dorsal tube (also called sessile haemocytes). To evaluate both haemocyte density, after collecting the haemolymph (for circulating cells), the fat body and adjacent organs were removed through the same incision to evaluate sessile cells. The fat body was collected in an Eppendorf tube containing 1 mL of chilled IPS, quickly vortexed, and centrifuged at 12,000 rpm, for 10 min at 4 ºC. The supernatant was removed from the tube, and the pellet was resuspended in 1 mL of cold IPS. The circulating and sessile haemocytes were counted using a Neubauer chamber. These experiments were performed using ISO (100 pM) and OCT (100 pM and 1 nM), and to validate the β-adrenergic origin of the responses elicited by them, we co-injected some larvae with ISO/OCT plus 10 µM of propranolol hydrochloride (Sigma, P8688), a β-blocker to try to blunt the responses [58].

### Direct influence of NE and ISO on *P. gingivalis* virulence and infection outcome

To assess the influence of ISO and NE directly on the virulence factors of *P. gingivalis*, bacteria were cultivated in broth with the presence of the ligands in the concentration that systemically influenced the haemocyte count *in vivo* (100 pM ISO, 1 µM NE) and with 100 µM (ISO and NE), a concentration that was shown to have activity in vitro [16–18]. The larvae were injected with PBS, *P. gingivalis* grown in PBS, *P. gingivalis* grown in 100 pM ISO (PgISO 100 pM) or 100 µM ISO (PgISO 100 µM) supplemented broth, *P. gingivalis* grown in 1 µM NE (PgNE 1 µM) or 100 µM NE (PgNE 100 µM) supplemented broth. After assessing that ISO increased *P. gingivalis* virulence (see Results section, Figure 9c,9b), in order to verify if the systemic administration of the ISO could influence the outcome when PgISO was administered, one more group was added: immediately after the injection of 100 pM ISO, *P. gingivalis* grown in 100 pM ISO was also administered (100 pM ISO + PgISO 100 pM). The larvae were followed hourly during 24 h, for survival assessment.

To investigate which genes were involved in the enhanced virulence of *P. gingivalis* grown with ISO or NE, we performed RT-qPCR. After the incubation period (24 h) the bacteria were centrifuged at 5,000 rpm for 10 min and the pellet frozen down at −80 °C. Later, we performed RNA extraction using TRIzol® following manufacturing instructions and sample quality, DNase treatment, transcription, amplification, reference gene selection (16S), and gene expression calculation were as described in section 5.10. The primers for the genes analysed are presented in Table 1.

### Statistical analysis

The experimental unit in this study was a single *G. mellonella* larvae, except for the *P. gingivalis* recovery assay in which a pool of three larvae was considered the experimental unit. Statistical analysis was carried out using the GraphPad Prism 6 program (GraphPad Software, LA Jolla California USA) or Orange [59] for creating the heatmaps. For haemocyte count, data were normalized by Z score, using the PBS group of each experiment as the baseline. After assessing the distribution, data were submitted to ANOVA or Kruskal-Wallis, *T*-test, or Mann-Whitney. Significance was assigned if p < 0.05.

**Figure 10.**
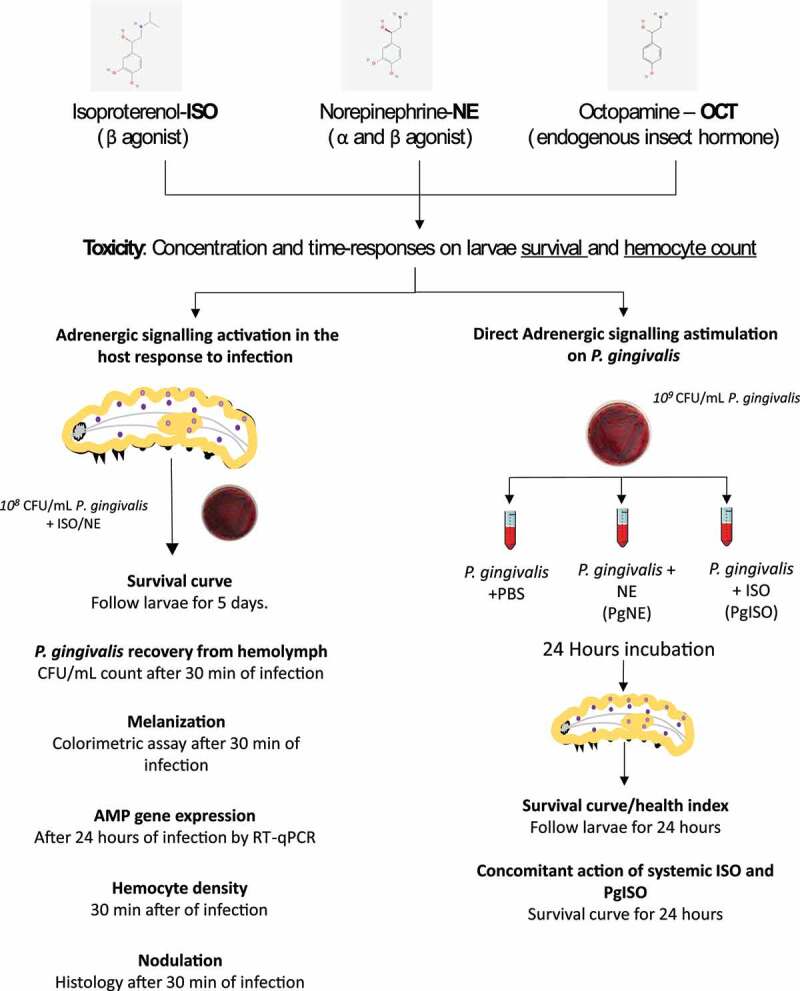
Direct action of β-adrenergic signalling on *P. gingivalis*. A) *rgbp* and *fimA* gene expression after co-culturing *P. gingivalis* +100pM or 100 μM ISO (rgbp, n = 6 biological replicates per group, mean and standard deviation, ANOVA, p < 0.0001; *fimA*, n=6 biological replicates per group, ANOVA, p = 0.0131) (a). Co-culturing *P. gingivalis* with 100 pM and 100 μM ISO increased the mortality of the larvae 24 h after infection (n = 10 larvae for PBS group, n = 45 larvae for *P. gingivalis* group, n = 20 for PgISO 100 pM and PgISO 100 μM and 100 pM ISO + PgISO 100 pM groups, Kaplan–Meier plot, log-rank [Mantel-cox] p < 0.0001) (b). The systemic administration of 100 pM ISO could partially reverse the increased PgISO 100pM phenotype (c). Same lower-case letters close to the graph bars/lines represent an absence of significant difference.

The study design described above is shown in Figure 10.

## Supplementary Material

Supplemental Material

## Data Availability

The data that support the findings of this study are openly available in the institutional repository at https://repositorio.unesp.br/handle/11449/234896
