# Supplementary material for: Effects of α and β-adrenergic signaling on innate immunity and Porphyromonas gingivalis virulence in an invertebrate model
Source: Virulence. 2022 Sep 19;13(1):1614–30. doi: 10.1080/21505594.2022.2123302 (PMC9487758; doi:10.1080/21505594.2022.2123302)
Supplement: Supplemental Material [file KVIR_A_2123302_SM8458.zip › supplementary/Supplementary legends.docx]

**Supplementary legends:**

**S1 appendix:** Octopamine (OCT) (A), norepinephrine (NE) (B), and isoproterenol (ISO) (C) concentration-response analysis of survival in *G. mellonella* larvae (Kaplan–Meyer plot, left) and health index (heatmaps, right). None of the three compounds showed significant toxicity (n = 20 larvae per group; Log-rank [Mantel-cox] test, OCT: p = 0.1313, NE: p = 0.6453, ISO: p = 0.7137). * Significantly different compared with the phosphate-buffered saline (PBS) group, using the Kruskal–Wallis test.

**S2 appendix:** Z-score-normalized hemocyte counts at 30 minutes post-injection of various concentrations of octopamine (OCT) in *G. mellonella* larvae. Box plots represent z-normalized hemocyte counts for n = 20 larvae per concentration. OCT increases the number of hemocytes at 100 pM and 1 nM (ANOVA, p < 0.0001). * Significantly different (p < 0.05) compared with the phosphate-buffered saline (PBS) group.

**S3 appendix:** Z-score-normalized hemocyte counts from hemolymph and fat body/adjacent organs in larvae that received PBS or 100pM/1nM octopamine (OCT) +/- propranolol [10µM] (n = 20 larvae per group, heatmap, ANOVA, p < 0.0001). * Significantly different compared with the PBS group (on hemolymph). Φ Significantly different compared with the PBS group (on fat body/adjacent organs).
