# Supplementary figures and images for: Effects of α and β-adrenergic signaling on innate immunity and Porphyromonas gingivalis virulence in an invertebrate model
Source: Virulence. 2022 Sep 19;13(1):1614–30. doi: 10.1080/21505594.2022.2123302 (PMC9487758; doi:10.1080/21505594.2022.2123302)

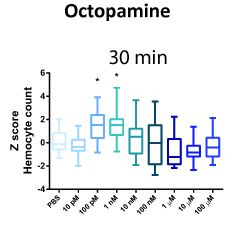

Supplement: Supplemental Material [file KVIR_A_2123302_SM8458.zip › supplementary/sup figure 2.tif]
